# Evolution reinforces cooperation with the emergence of self-recognition mechanisms: An empirical study of strategies in the Moran process for the iterated prisoner’s dilemma

**DOI:** 10.1371/journal.pone.0204981

**Published:** 2018-10-25

**Authors:** Vincent Knight, Marc Harper, Nikoleta E. Glynatsi, Owen Campbell

**Affiliations:** 1 Cardiff University, School of Mathematics, Cardiff, United Kingdom; 2 Google Inc., Mountain View, CA, United States of America; 3 Independent Researcher, Chester, United Kingdom; Peking University, CHINA

## Abstract

We present insights and empirical results from an extensive numerical study of the evolutionary dynamics of the iterated prisoner’s dilemma. Fixation probabilities for Moran processes are obtained for all pairs of 164 different strategies including classics such as TitForTat, zero determinant strategies, and many more sophisticated strategies. Players with long memories and sophisticated behaviours outperform many strategies that perform well in a two player setting. Moreover we introduce several strategies trained with evolutionary algorithms to excel at the Moran process. These strategies are excellent invaders and resistors of invasion and in some cases naturally evolve handshaking mechanisms to resist invasion. The best invaders were those trained to maximize total payoff while the best resistors invoke handshake mechanisms. This suggests that while maximizing individual payoff can lead to the evolution of cooperation through invasion, the relatively weak invasion resistance of payoff maximizing strategies are not as evolutionarily stable as strategies employing handshake mechanisms.

## Introduction

The Prisoner’s Dilemma (PD) [1] is a fundamental two player game used to model a variety of strategic interactions. Each player chooses simultaneously and independently between cooperation (C) or defection (D). The payoffs of the game are defined by the matrix $\left(\begin{array}{cc} R & S\\ T & P \end{array}\right)$, where *T* > *R* > *P* > *S* and 2*R* > *T* + *S*. The PD is a one round game, but is commonly studied in a manner where the prior outcomes matter. This repeated form is called the Iterated Prisoner’s Dilemma (IPD). As described in [2–4] a number of strategies have been developed to take advantage of the history of play. Strategies referred to as zero determinant (ZD) strategies [4] can manipulate some players through extortionate mechanisms.

The Moran Process [5] is a model of evolutionary population dynamics that has been used to gain insights about the evolutionary stability in a number of settings. Several earlier works have studied iterated games in the context of the prisoner’s dilemma [6, 7], however these often make simplifying assumptions or are limited to classes of strategies such as memory-one strategies that only use the previous round of play.

This manuscript provides a detailed numerical analysis of agent-based simulations of **164** complex and adaptive strategies for the IPD. This is made possible by the Axelrod library [8], an effort to provide software for reproducible research for the IPD. The library now contains over 186 parameterized strategies including classics like TitForTat and WinStayLoseShift, as well as recent variants such as OmegaTFT, zero determinant and other memory one strategies, strategies based on finite state machines, lookup tables, neural networks, and other machine learning based strategies, and a collection of novel strategies. Not all strategies have been considered for this study: excluded are those that make use of knowledge of the number of turns in a match and others that have a high computational run time. The large number of strategies are available thanks to the open source nature of the project with over 50 contributors from around the world, made by programmers and researchers [3]. Three of the considered strategies are finite state machines trained specifically for Moran processes (described further in the Methods section).

In addition to providing a large collection of strategies, the Axelrod library can conduct matches, tournaments and population dynamics with variations including noise and spatial structure. The strategies and simulation frameworks are automatically tested to an extraordinarily high degree of coverage in accordance with best research software practices.

Using the Axelrod library and the many strategies it contains, we obtain the probability with which a given strategy takes over a population (referred to as fixation probability) for all pairs of strategies, identifying those that are effective invaders and those resistant to invasion, for population sizes *N* = 2 to *N* = 14. Moreover we present a number (16) of strategies that were created via reinforcement algorithms (evolutionary and particle swarm algorithms) that are among the best invaders and resistors of invasion known to date, and show that handshaking mechanisms naturally arise from these processes as an invasion-resistance mechanism.

In 2016, work has argued that agent-based simulations can provide insights in evolutionary game theory not available via direct mathematical analysis [9]. The results and insights contained in this paper would be difficult to derive analytically.

In particular the following questions are addressed:

1. What strategies are good invaders?
2. What strategies are good at resisting invasion?
3. How does the population size affect these findings?

While the results agree with some of the published literature, it is found that:

1. Zero determinant strategies are not effective invaders or defenders for *N* > 2.
2. Complex strategies can be effective, and in fact can naturally evolve through evolutionary processes to outperform designed strategies.
3. The strongest resistors specifically evolve or possess a handshake mechanism.
4. Strong invaders are generally cooperative strategies that do not defect first but retaliate to varying degrees of intensity against strategies that defect.
5. Strategies evolved to maximize their total payoff can be strong invaders and achieve mutual cooperation with many other strategies.

The notion of a **handshake** has been described previously in [10] and corresponds to the idea that an individual exhibits behaviour that starts with a recognisable pattern. This in turn allows them to identify individuals of their own type (who would exhibit the same pattern). This is analogous to the biological notion of ‘kin recognition’ where individuals have the ability to recognise the phenotype of their own kin [11].

## Materials and methods

### The Moran process

**Fig 1:**
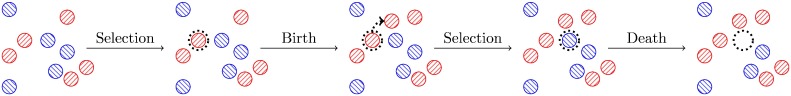
A diagrammatic representation of a Moran process.

A Moran process is a stochastic birth death process on a finite population in which the population size stays constant over time. Individuals are *selected* according to a given fitness landscape. The fitness landscape in this work is defined as the total utility against all other individuals in the population. Once selected, the individual is reproduced and similarly another individual is chosen to be removed from the population (a uniform random selection is used). This is shown diagrammatically in Fig 1. In some settings mutation is also considered but without mutation (the case considered in this work) this process will arrive at an absorbing state where the population is entirely made up of players of one strategy. The probability with which a given strategy, starting from a single individual takes over a population is called the *fixation probability*. A more detailed analytic description of this is given later. In our simulations offspring do not inherit any knowledge or history from parent replicants.

The Moran process was initially introduced in [5]. It has since been used in a variety of settings including the understanding of the spread of cooperative and non-cooperative behaviour such as cancer [12] and the emergence of cooperative behaviour in spatial topologies [13]. However these works mainly consider relatively simple strategies. A few works looked at evolutionary stability of agent-based strategies within the Prisoner’s Dilemma [14] but this is not done in the more widely used setting of the Moran process, rather in terms of infinite population stability. In [15] Moran processes are studied in a theoretical framework for a small subset of strategies. The subset included memory one strategies: strategies that recall the events of the previous round only.

Of particular interest are the zero determinant strategies introduced in [4]. It was argued in [7] that generous ZD strategies are robust against invading strategies. However, in [16], a strategy using machine learning techniques was capable of resisting invasion and also able to invade any memory one strategy. In 2017, [17] has investigated the effect of memory length on strategy performance and the emergence of cooperation but this is not done in a Moran process context and only considers specific cases of memory 2 strategies. In [18] it was recognised that many zero determinant strategies do not fare well against themselves. This is a disadvantage for the Moran process where the best strategies cooperate well with other players using the same strategy.

This work uses pair-wise Moran processes in a similar way to matches in the many IPD tournaments published since Axelrod’s original work [2]. A population-based perspective is given which adds additional evolutionary components to the IPD, namely the evolutionary dynamics of invasion and resistance.

### Strategies considered

To carry out this numerical experiment, 164 strategies, listed (with their properties) in the Appendix, are used from the Axelrod library. The appendix also includes citations to the original description of each strategy. There are 43 stochastic and 121 deterministic strategies. Their memory depth, defined by the number of rounds of history used by the strategy each round, is shown in Table 1. The memory depth is infinite if the strategy uses the entire history of play (whatever its length). For example, a strategy that utilizes a handshaking mechanism where the opponent’s actions on the first few rounds of play determines the strategies subsequent behavior would have infinite memory depth.

**Table 1:** Memory depths.

Memory Deptd	0	1	2	3	4	5	6	9	10	11	12	16	20	40	200	∞
Count	3	29	12	8	2	6	1	1	5	1	1	2	2	2	1	88

Using families of strategies that depend on given parameters it is possible to find specific parameters through a training process called reinforcement learning. A detailed description of the various types considered is given in [19].

A number of these strategies have been trained this way (see [19]) prior to this study and not specifically for the Moran process. For example:

- Evolved ANN: a neural network based strategy;
- Evolved LookerUp: a lookup table based strategy;
- PSO Gambler: a stochastic version of the lookup table based strategy;
- Evolved HMM: a hidden Markov model based strategy.

Apart from the PSO Gambler strategy, which was trained using a particle swarm optimisation algorithm, these strategies are trained with an evolutionary algorithm that perturbs strategy parameters and optimizes the mean total score against all other opponents [20]. They were trained to win IPD tournaments by maximizing their mean total payoffs against a variety of opponents. Variation is introduced via mutation and crossover of parameters, and the best performing strategies are carried to the next generation along with new variants. Similar methods appear in the literature [21]. There has also been some work on strategies using an evolutionary algorithm in real time: in [22] an evolutionary algorithm is used to build a model of the opponent and attempt to exploit any potential weakness. In this work all strategies resulting from evolutionary algorithms are pre-trained.

More information about each player can be obtained in the documentation for [8] and a detailed description of the performance of these strategies in IPD tournaments is described in [19].

All of the training code is archived at [23]. This software is (similarly to the Axelrod library) available on GitHub (https://github.com/Axelrod-Python/axelrod-dojo) with documentation to train new strategies easily. Training typically takes less than 100 generations and can be completed within several hours on commodity hardware.

One particular family of strategies that has been studied in the literature are called finite state machines. These mathematical models consist of states and responses to actions which indicate a next state given an action. In the context of the IPD, a finite state machine, is a mapping from an arbitrary list of states and opponent actions (cooperation or defection) to states and an action (cooperation or defection). For further details, the reader is referred to [19, 21, 24, 25].

**Fig 2:**
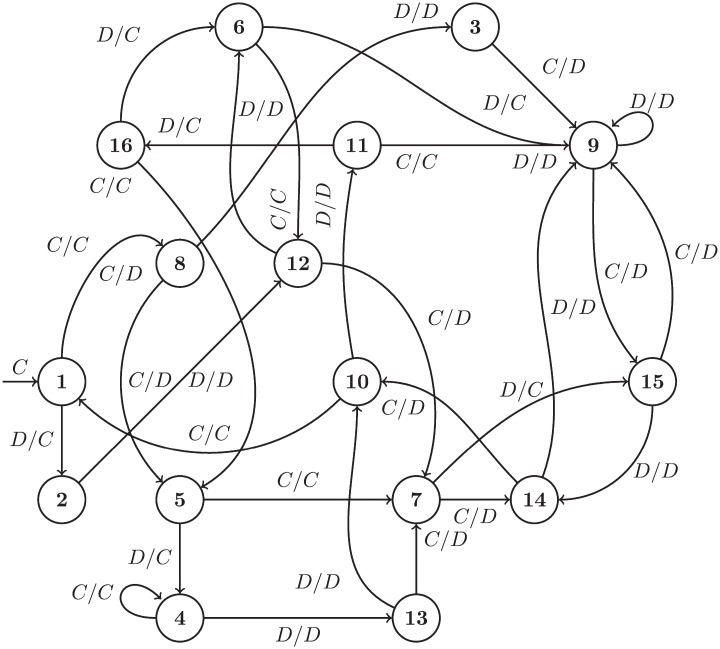
TF1: A 16 state finite state machine with a handshake leading to mutual cooperation at state 4.

**Fig 3:**
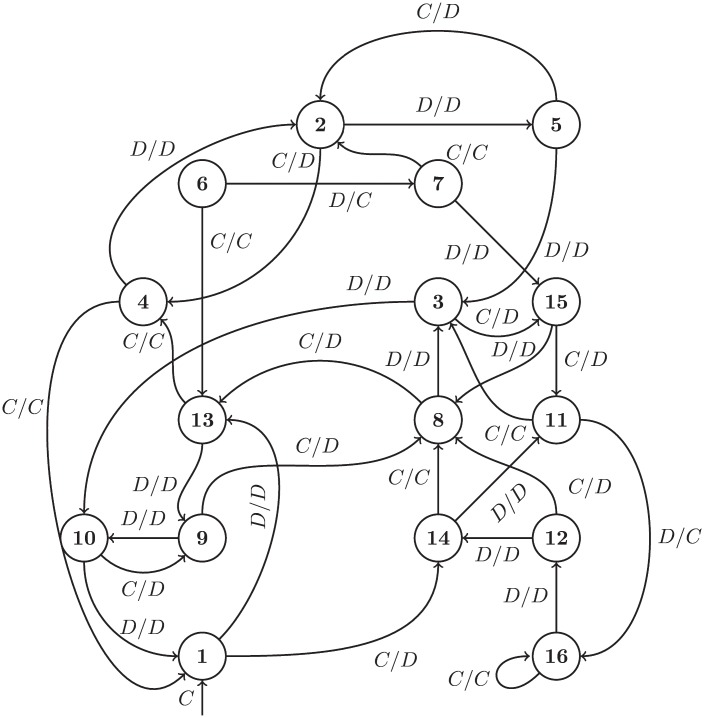
TF2: A 16 state finite state machine with a handshake leading to mutual cooperation at state 16.

**Fig 4:**
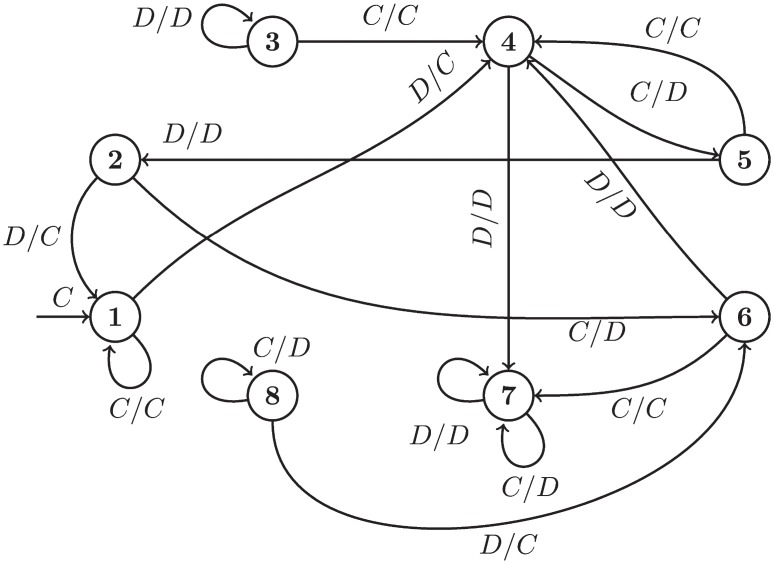
TF3: An 8 state finite state machine.

There are three further strategies trained specifically for this study; Trained FSM 1, 2, and 3 (TF1 TF3). These are finite state machines of 16, 16, and 8 states respectively. These are shown in Figs 2, 3 and 4, using the notation common in the literature where *A*_1_/*A*_2_ is the action of the opponent *A*_1_ and the response of the player *A*_2_ as well as arrows corresponding to changes of state.

As opposed to the previously described strategies [19], these strategies were trained with the objective function of *mean fixation probabilities for Moran processes* starting at initial population states consisting of *N*/2 individuals of the training candidates and *N*/2 individuals of an opponent strategy, taken from a selection of 150 opponents from the Axelrod library:

- TF1 *N* = 12, 0% noise.
- TF2 *N* = 10, 0% noise.
- TF3 *N* = 8, 1% noise.

The trained algorithms were run for fewer than 50 generations. Training data for this is available at [26].

TF1 has an initial handshake of CCD and cooperates if the opponent matches. However if the opponent later defects, TF1 will respond in kind, so the handshake is not permanent. Only one player (Prober 4 [27]) manages to achieve cooperation with TF1 after about 20 rounds of play. TF1 is functionally very similar to a strategy known as “Collective Strategy”, which has a handshake of CD and cooperates with opponents that matched the handshake until they defect, defecting thereafter if the opponent ever defects [28]. Collective Strategy was specifically designed for evolutionary processes.

TF2 always starts with CD and will defect against opponents that start with DD. It plays CDD against itself and then cooperates thereafter; Fortress3 and Fortress4 also use a similar handshake and cooperate with TF2. Cooperation can be rescued after a failed handshake by a complex sequence of plays which sometimes results in mutual cooperation with Firm but Fair, Grofman, and GTFT, and a few others with low probability. TF2 defects against all other players in the study, barring unusual cases arising from particular randomizations. Fig 3 shows all 16 states of the strategy (states 6 and 7 are not reachable).

TF3 cooperates and defects with various cycles depending on the opponent’s actions. TF3 will mutually cooperate with any strategy and only tolerates a few defections before defecting for the rest of match. It is similar to but not exactly the same as Fool Me Once, a strategy that cooperates until the opponent has defected twice (not necessarily consecutively), and defects indefinitely thereafter. Though a product of training with a Moran objective, it differs from TF1 and TF2 in that it lacks a handshake mechanism. Fig 4 shows all 8 states of the strategy produced by the training process (states 3 and 8 are not reachable).

For both TF1 and TF2 a handshake mechanism naturally emerges from the structure of the underlying finite state machine. This behavior is an outcome of the evolutionary process and is in no way hard-coded or included via an additional mechanism.

### Data collection

Each strategy pair is run for 1000 repetitions of the Moran process to fixation with starting population distributions of (1, *N* − 1), (*N*/2, *N*/2) and (*N* − 1, 1), for *N* from 2 through 14. The fixation probability is then empirically computed for each combination of starting distribution and value of *N*. The Axelrod library can carry out exact simulations of the Moran process. Since some of the strategies have a high computational cost or are stochastic, samples are taken from a large number of 200 turn match outcomes for the pairs of players for use in computing fitnesses in the Moran process (i.e. a stochastic cache of matches is used). This approach was verified to agree with unsampled calculations to a high degree of accuracy in specific cases. This is described in Algorithms 1 and 2.

**Algorithm 1** Data Collection

1: **for** player_one **in** players_list **do**

2:  **for** player_two **in** (players_list—player_one) pair **do**

3:   pair ← (player_one, player_two)

4:   **for** starting_population_distributions **in** [$\left(1,N-1\right),\left(\frac{N}{2},\frac{N}{2}\right),\left(N-1,1\right)]$ **do**

5:    **while** repetitions ≤ 1000 **do**

6:     **simulate** moran process (pair, starting distribution)

7:    **end while**

8:    **yield** fixation probabilities

9:   **end for**

10:  **end for**

11: **end for**

**Algorithm 2** Moran process

1: initial population ← (pair, starting distribution)

2: population ← initial population

3: **while** population not uniform **do**

4:  **for** player in population **do**

5:   **for** opponent in (population—player) **do**

6:    match ← (player, opponent)

7:    results ← stochastic_cache (200 round match)

8:   **end for**

9:  **end for**

10:  population ← sorted(results)

11:  parent ← selected randomly in proportion to its total match payoffs

12:  offspring ← parent

13:  kill off ← uniformly random player from population

14:  population ← offspring replaces kill off

15: **end while**

The next section will further validate the methodology by comparing simulated results to analytical results in a few selected cases. The main results of this manuscript will present a detailed analysis of all the data generated. Finally, a discussion and conclusion will offer future avenues for the work presented here.

## Results

### Validation

As described in [6] consider the payoff matrix:

$$\begin{array}{c} M=(\begin{array}{c} a,b\\ c,d \end{array}) \end{array}$$ (1)

The expected payoffs of *i* players of the first type in a population with *N* − *i* players of the second type are given by:

$$\begin{array}{c} f_{i}=\frac{a(i-1)+b(N-i)}{N-1} \end{array}$$ (2)

$$\begin{array}{c} g_{i}=\frac{ci+d(N-i-1)}{N-1} \end{array}$$ (3)

The transitions within the birth death process that underpins the Moran process are then given by:

$$p_{i,i+1}=\frac{if_{i}}{if_{i}+\left(N-i\right)g_{i}}\frac{N-i}{N}$$ (4)

$$p_{i,i-1}=\frac{\left(N-i\right)g_{i}}{if_{i}+\left(N-i\right)g_{i}}\frac{i}{N}$$ (5)

$$p_{ii}=1-p_{i,i+1}-p_{i,i-1}$$ (6)

Using this the fixation probability of the first strategy in a population of *i* individuals of the first type and *N* − *i* individuals of the second, is given by [13]:

$$\begin{array}{c} x_{i}=\frac{1+\sum_{j=1}^{i-1}\prod_{k=1}^{j}\gamma_{j}}{1+\sum_{j=1}^{N-1}\prod_{k=1}^{j}\gamma_{j}} \end{array}$$ (7)

where:

$$\gamma_{j}=\frac{p_{j,j-1}}{p_{j,j+1}}$$

A neutral strategy will have fixation probability *x*_*i*_ = *i*/*N*.

**Fig 5:**
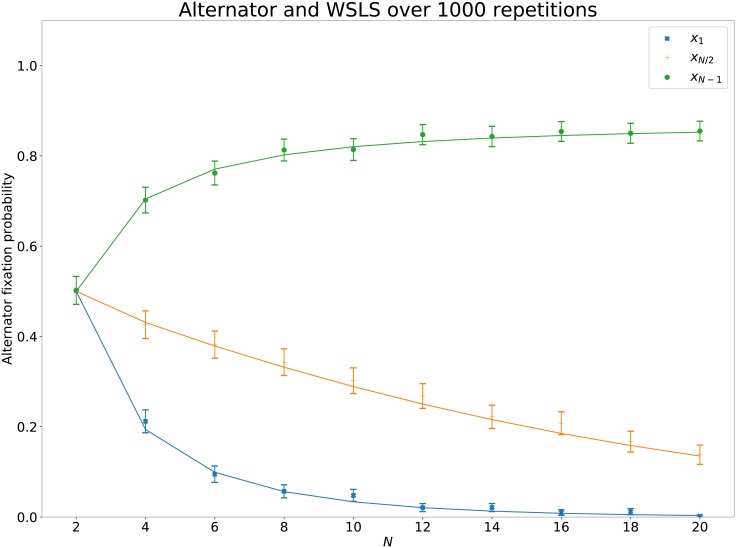
Comparison of theoretic and actual Moran process fixation probabilities for *deterministic* strategies: Alternator and Cooperator. 5% confidence intervals calculated using an asymptotic normal approximation. The top most line on all figures (in red and using a circle) corresponds to *x*_*N*−1_, the middle line (in green and using a cross) corresponds to *x*_*N*/2_ and the bottom line (in blue and using an x) corresponds to *x*_1_.

Comparisons of *x*_1_, *x*_*N*/2_, *x*_*N*−1_ are shown in Fig 5 for Alternator and WSLS (a 5% confidence interval computed using an asymptotic normal approximation is also included [29]). The points represent the simulated values and the line shows the theoretical value. Note that these are deterministic strategies and show a good match between the expected value of (7) and the actual Moran process for all strategy pairs. These means have been compared using a *t*-test and the *p* values are shown in Table 2 which confirms the fact that the theoretic and simulated values are a good match.

**Table 2:** *p* values resulting from a *t* test comparing the theoretic value with the simulated value of the Moran process fixation probabilities for *deterministic* strategies: Alternator and Cooperator.

N	*p*_1_ *p* Value	*p*_*N*/2_ *p* Value	*p*_*N*−1_ *p* Value
2	0.89942	0.89942	0.89942
4	0.16344	0.74768	0.85877
6	0.65406	0.84051	0.51992
8	0.92617	0.45748	0.38604
10	0.03505	0.37115	0.60635
12	0.96697	0.20790	0.18124
14	0.08126	0.63771	0.76246
16	0.59434	0.07697	0.42520
18	0.05339	0.47126	0.94814
20	0.30805	0.79795	0.82381

**Fig 6:**
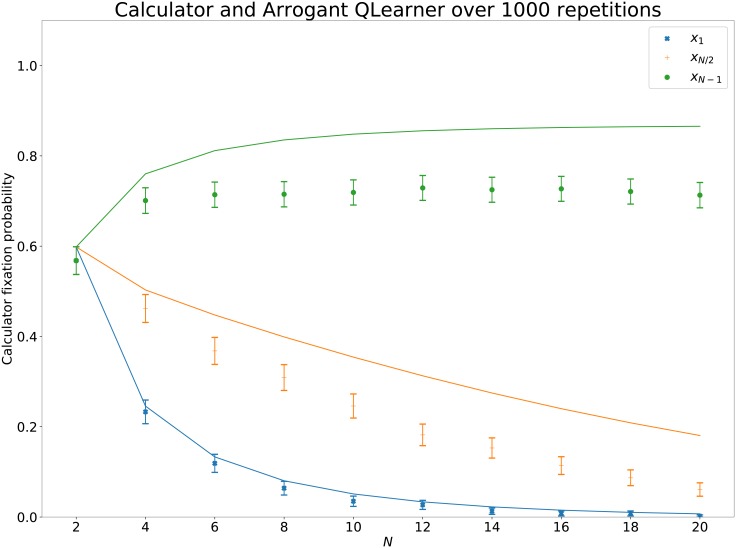
Comparison of theoretic and actual Moran process fixation probabilities for *stochastic* strategies: Calculator and Arrogant Q Learner. 5% confidence intervals calculated using an asymptotic normal approximation. The top most line on all figures (in red and using a circle) corresponds to *x*_*N*−1_, the middle line (in green and using a cross) corresponds to *x*_*N*/2_ and the bottom line (in blue and using an x) corresponds to *x*_1_.

Fig 6 shows the fixation probabilities for stochastic strategies: Calculator and arrogant Q Learner. These are no longer a good match (confirmed with a *t*-test in Table 3). This demonstrates that assuming a given interaction between two IPD strategies can be summarised with a set of utilities as shown in (1) is not correct. For any given pair of strategies it is possible to obtain *p*_*i*, *i*−1_, *p*_*i*, *i*+ 1_, *p*_*ii*_ exactly (as opposed to the approximations offered by (4), (5) and (6)). Obtaining these requires particular analysis for a given pair and can be quite a complex endeavour for stochastic strategies with long memory: this is not necessary for the purposes of this work. All data generated for this validation exercise can be found at [26].

**Table 3:** *p* − values resulting from a *t* − test comparing the theoretic value with the simulated value of the Moran process fixation probabilities for *stochastic* strategies: Calculator and Arrogant Q Learner.

N	*p*_1_ *p* Value	*p*_*N*/2_ *p* Value	*p*_*N*−1_ *p* Value
2	0.05372	0.05372	0.05372
4	0.34188	0.00940	0.00005
6	0.16510	0.00000	0.00000
8	0.03451	0.00000	0.00000
10	0.00543	0.00000	0.00000
12	0.19646	0.00000	0.00000
14	0.00814	0.00000	0.00000
16	0.01038	0.00000	0.00000
18	0.39556	0.00000	0.00000
20	0.00030	0.00000	0.00000

### Empirical results

This section outlines the data analysis carried out, all data for this study is available at [26].

- First the specific case of *N* = 2 is considered.
- The effect of population size on the ability of a strategy to invade another population is investigated. This will highlight how complex strategies with long memories outperform simpler strategies.
- Then a similar investigation of the ability to defend against an invasion is given.
- Finally the relationship between performance for differing population sizes as well as taking a close look at zero determinant strategies [4] is analysed.

#### The special case of *N* = 2

When *N* = 2 the fixation probabilities of the Moran process are effectively measures of the distribution of relative mean payoffs over all possible matches between two players. The strategy that scores higher than the other more often will fixate more often. For *N* = 2 the two cases of *x*_1_ and *x*_*N*−1_ coincide, but will be considered separately for larger *N* in the following sections. The top 16 (10%) strategies are shown in Table 4 and figures showing the performance of all strategies are available in the appendix. The top five ranking strategies are:

1. The top strategy is the Collective Strategy (CS) which has a simple handshake mechanism described above.
2. Defector: it always defects. Since it has no interactions with other defectors (recall that *N* = 2), its aggressiveness is rewarded.
3. Aggravater, which plays like Grudger (responding to any defections with unconditional defections throughout) however starts by playing 3 defections.
4. Predator, a finite state machine described in [21].
5. Handshake, a slightly less aggressive version of the Collective Strategy [10]. As long as the initial sequence is played then it cooperates. Thus it will do well in a population consisting of many members of itself just as the Collective Strategy does. The difference is that CS will defect after the handshake if the opponent defects while Handshake will not.

**Table 4:** Top strategies for *N* = 2 (neutral fixation is *p* = 0.5).

	Player	Min	5th %	Mean	Median	95th %	Max	Std
1	CS	0.497	0.5020	0.6651	0.572	0.9908	0.993	0.1800
2	Defector	0.502	0.5020	0.6496	0.518	1.0000	1.000	0.1767
3	Aggravater	0.502	0.5020	0.6328	0.518	0.9790	0.999	0.1660
4	Predator	0.319	0.4980	0.6301	0.551	0.9870	0.993	0.1676
5	Handshake	0.006	0.4503	0.6240	0.524	0.9908	0.993	0.1889
6	Prober 4	0.431	0.4620	0.6183	0.534	0.9579	0.958	0.1656
7	**TF1**	0.430	0.4980	0.6171	0.544	0.9240	0.981	0.1345
8	Prober 3	0.497	0.5011	0.6044	0.505	0.9930	0.993	0.1683
9	**TF2**	0.324	0.5020	0.6026	0.565	0.8362	0.887	0.1092
10	Grudger	0.497	0.5020	0.5996	0.502	0.9840	0.989	0.1695
11	Better and Better	0.388	0.3951	0.5980	0.514	0.9300	0.934	0.1865
12	MEM2	0.497	0.5001	0.5942	0.502	0.9840	0.987	0.1656
13	Meta Hunter Aggressive	0.247	0.2943	0.5933	0.517	0.9498	0.981	0.2013
14	**TF3**	0.344	0.4971	0.5927	0.502	0.9790	0.982	0.1617
15	Fool Me Once	0.494	0.4980	0.5892	0.502	0.9790	0.982	0.1625
16	ZD-Extort-4	0.497	0.5020	0.5867	0.584	0.6900	0.695	0.0724

It is also noted that TF1, TF2 and TF3 all perform well for this case of *N* = 2. This is also the value of *N* for which a zero determinant strategy does appear in the top 10% ranking strategies: ZD-extort-4. The performance of zero determinant strategies will be examined more closely.

As will be demonstrated the results for *N* = 2 differ from those of larger *N*. Hence these results do not concur with the literature which suggests that zero determinant strategies should be effective for larger population sizes, but these analyses consider stationary behaviour, while this work runs for a fixed number of rounds [7]. The stationarity assumption allows for a deterministic payoff matrix leading to the conclusions about zero determinant strategies in the space of memory-one strategies that do not generalize to this context.

#### Strong invaders

**Fig 7:**
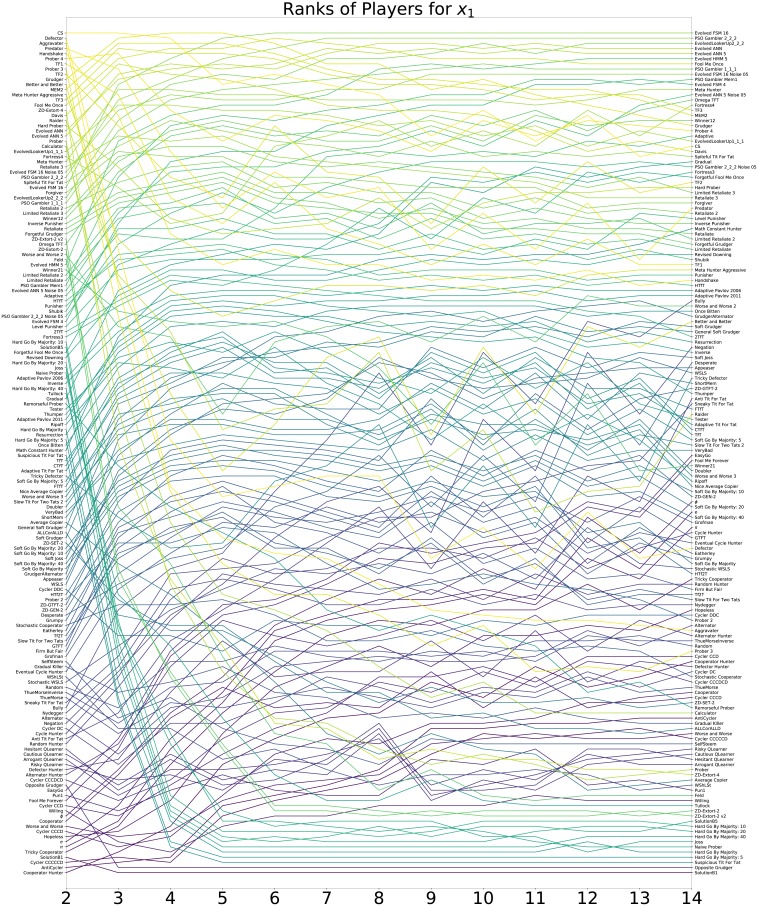
Invasion: Ranks of all strategies according to *x*_1_ for different population sizes.

In this section the focus is on the ability of a mutant strategy to invade: the probability of one individual of a given type successfully fixating in a population of *N*−1 other individuals, denoted by *x*_1_. The ranks of each strategy for all considered values of *N* according to mean *x*_1_ are shown in Fig 7.

The top 16 strategies are given in Tables 5, 6 and 7. A variety of figures showing the performance of all strategies is available in the supporting information.

**Table 5:** Top invaders for *N* = 3.

	Player	Min	5th %	Mean	Median	95th %	Max	Std
1	CS	0.261	0.2620	0.4478	0.403	0.8105	0.908	0.1998
2	Grudger	0.259	0.2641	0.4313	0.338	0.8097	0.908	0.1699
3	MEM2	0.258	0.2875	0.4278	0.338	0.7977	0.907	0.1636
4	**TF3**	0.248	0.2610	0.4267	0.338	0.7904	0.904	0.1624
5	Prober 4	0.221	0.2400	0.4242	0.365	0.7723	0.891	0.1755
6	Fool Me Once	0.257	0.2645	0.4242	0.338	0.7938	0.904	0.1620
7	Davis	0.234	0.2581	0.4218	0.338	0.7759	0.891	0.1590
8	Predator	0.173	0.2590	0.4210	0.374	0.7845	0.907	0.1824
9	Evolved ANN 5	0.255	0.3163	0.4163	0.338	0.7872	0.879	0.1530
10	Evolved ANN	0.253	0.2789	0.4163	0.338	0.7938	0.906	0.1572
11	Evolved FSM 16	0.041	0.1391	0.4154	0.338	0.7977	0.907	0.1830
12	Meta Hunter	0.123	0.2541	0.4140	0.338	0.7807	0.892	0.1614
13	**TF1**	0.257	0.2580	0.4139	0.398	0.7411	0.900	0.1529
14	PSO Gambler 2_2_2	0.073	0.2643	0.4134	0.338	0.7938	0.904	0.1727
15	EvolvedLookerUp1_1_1	0.258	0.3004	0.4113	0.338	0.7515	0.830	0.1369
16	Evolved FSM 16 Noise 05	0.247	0.3238	0.4107	0.338	0.7977	0.906	0.1540

**Table 6:** Top invaders for *N* = 7.

	Player	Min	5th %	Mean	Median	95th %	Max	Std
1	Evolved FSM 16	0.001	0.0313	0.2523	0.142	0.6389	0.826	0.1931
2	PSO Gambler 2_2_2	0.004	0.0588	0.2467	0.142	0.6096	0.826	0.1809
3	Fool Me Once	0.044	0.0470	0.2459	0.142	0.6105	0.826	0.1792
4	Evolved ANN 5	0.044	0.1092	0.2450	0.142	0.6010	0.812	0.1722
5	Evolved ANN	0.042	0.0615	0.2449	0.142	0.6104	0.826	0.1785
6	EvolvedLookerUp2_2_2	0.000	0.0618	0.2443	0.142	0.6380	0.824	0.1822
7	Grudger	0.044	0.0451	0.2442	0.142	0.6420	0.826	0.1830
8	MEM2	0.044	0.0583	0.2436	0.142	0.6143	0.826	0.1760
9	**TF3**	0.044	0.0450	0.2430	0.142	0.6344	0.826	0.1779
10	PSO Gambler 1_1_1	0.021	0.1033	0.2404	0.142	0.6381	0.824	0.1710
11	CS	0.045	0.0450	0.2395	0.148	0.6385	0.826	0.2169
12	Evolved FSM 16 Noise 05	0.044	0.1298	0.2394	0.142	0.6143	0.826	0.1732
13	Evolved HMM 5	0.010	0.0611	0.2390	0.142	0.6115	0.826	0.1785
14	Meta Hunter	0.015	0.0465	0.2385	0.142	0.5993	0.820	0.1751
15	Davis	0.036	0.0465	0.2379	0.142	0.5953	0.820	0.1732
16	PSO Gambler Mem1	0.018	0.1105	0.2348	0.142	0.6370	0.825	0.1671

**Table 7:** Top invaders for *N* = 14.

	Player	Min	5th %	Mean	Median	95th %	Max	Std
1	Evolved FSM 16	0.000	0.0054	0.2096	0.079	0.7241	0.842	0.2172
2	PSO Gambler 2_2_2	0.000	0.0113	0.2042	0.079	0.5940	0.842	0.2045
3	EvolvedLookerUp2_2_2	0.000	0.0270	0.2014	0.079	0.6608	0.840	0.2097
4	Evolved ANN	0.002	0.0164	0.2014	0.079	0.5939	0.842	0.2074
5	Evolved ANN 5	0.002	0.0505	0.2004	0.079	0.5940	0.834	0.2009
6	Evolved HMM 5	0.000	0.0321	0.1972	0.079	0.5940	0.842	0.2034
7	PSO Gambler 1_1_1	0.001	0.0455	0.1955	0.079	0.6150	0.841	0.1931
8	Fool Me Once	0.002	0.0058	0.1955	0.079	0.5940	0.842	0.2032
9	Evolved FSM 16 Noise 05	0.003	0.0607	0.1943	0.079	0.5930	0.842	0.2005
10	PSO Gambler Mem1	0.000	0.0517	0.1920	0.079	0.6118	0.841	0.1907
11	Evolved FSM 4	0.000	0.0000	0.1918	0.079	0.5930	0.842	0.2049
12	Meta Hunter	0.000	0.0049	0.1869	0.079	0.5883	0.840	0.1882
13	Evolved ANN 5 Noise 05	0.001	0.0303	0.1858	0.079	0.5930	0.840	0.1968
14	Omega TFT	0.003	0.0704	0.1849	0.079	0.5939	0.840	0.1927
15	Fortress4	0.000	0.0000	0.1848	0.066	0.5919	0.840	0.2211
16	**TF3**	0.002	0.0041	0.1846	0.079	0.6190	0.842	0.1890

It can be seen that apart from CS, none of the strategies for *N* = 2 of Table 4 perform well for *N* ∈ {3, 7, 14}. The new top performing strategies are:

- Grudger (which only performs well for *N* = 3), starts by cooperating but will defect if at any point the opponent has defected.
- MEM2, an infinite memory strategy that switches between TFT, TF2T, and Defector [14].
- TF3, the finite state machine trained specifically for Moran processes described.
- Prober 4, a strategy which starts with a specific 20 move sequence of cooperations and defections [27]. This initial sequence serves as approximate handshake.
- PSO Gambler and Evolved Lookerup 2 2 2: strategies that make use of a lookup table mapping the first 2 moves of the opponent as well as the last 2 moves of both players to an action. PSO gambler is a stochastic version of Lookerup which maps those states to probabilities of cooperating. Lookerup was described in [3].
- The Evolved ANN strategies are neural networks that map a number of attributes (first move, number of cooperations, last move, etc.) to an action. Both of these have been trained using an evolutionary algorithm.
- Evolved FSM 16 is a 16 state finite state machine trained to perform well in tournaments.

Only one of the above strategies is stochastic although close inspection of the source code of PSO Gambler shows that it makes stochastic decisions rarely, and is functionally very similar to its deterministic cousin Evolved Looker Up. PSO Gambler Mem1 is a stochastic memory one strategy that has been trained to maximise its utility and does perform well. Apart from TF3, the finite state machines trained specifically for Moran processes do not appear in the top 5, while strategies trained for tournaments do. This is due to the nature of invasion: most of the opponents will initially be different strategies. The next section will consider the converse situation.

#### Strong resistors

**Fig 8:**
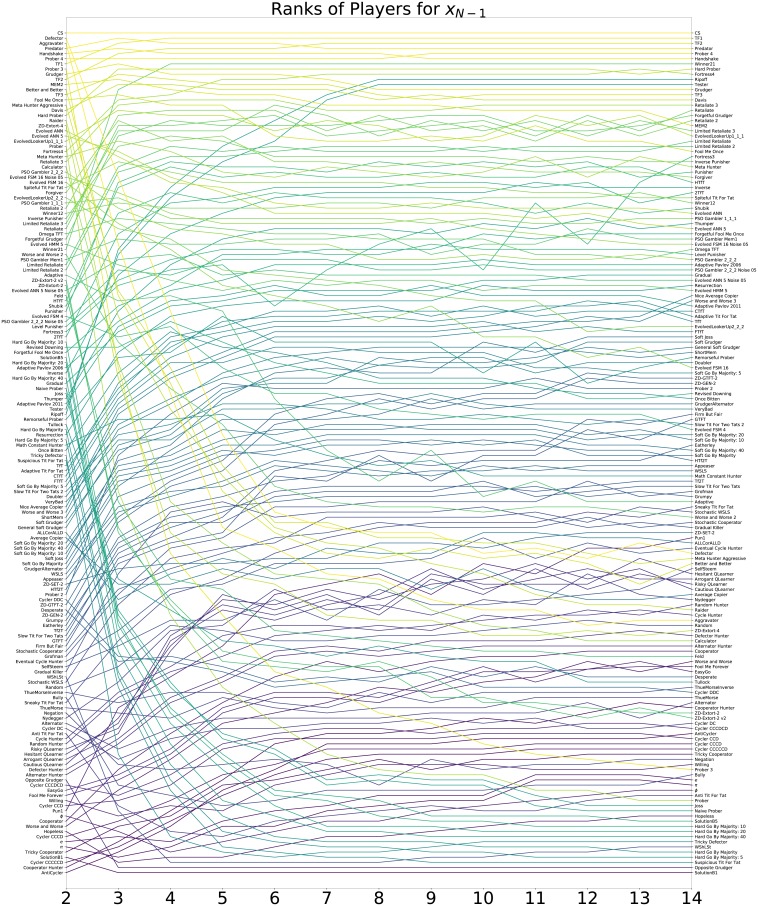
Resistance: Ranks of all strategies according to *x*_*N*−1_ for different population sizes.

In addition to identifying good invaders, strategies resistant to invasion by other strategies are identified by examining the distribution of *x*_*N*−1_ for each strategy. The ranks of each strategy for all considered values of *N* according to mean *x*_*N*−1_ are shown in Fig 8.

Tables 8, 9 and 10 show the top strategies when ranked according to *x*_*N*−1_ for *N* ∈ {3, 7, 14} and figures showing results for all strategies are available in the supplementary materials. Once again none of the short memory strategies previously discussed perform well for high *N*.

**Table 8:** Top resistors for *N* = 3.

	Player	Min	5th %	Mean	Median	95th %	Max	Std
1	CS	0.662	0.7410	0.8359	0.796	0.9980	1.000	0.0981
2	Predator	0.530	0.7363	0.8121	0.789	0.9980	1.000	0.0983
3	**TF1**	0.648	0.7330	0.8087	0.791	0.9745	0.999	0.0775
4	Handshake	0.225	0.6322	0.8014	0.779	0.9980	1.000	0.1293
5	**TF2**	0.572	0.7363	0.7957	0.790	0.9330	0.961	0.0672
6	Prober 4	0.646	0.6610	0.7905	0.750	0.9890	0.996	0.1070
7	Grudger	0.662	0.6620	0.7612	0.662	0.9980	1.000	0.1224
8	Hard Prober	0.661	0.6620	0.7582	0.732	0.9980	0.999	0.1079
9	**TF3**	0.594	0.6620	0.7570	0.662	0.9969	0.999	0.1197
10	MEM2	0.662	0.6620	0.7554	0.662	0.9980	1.000	0.1210
11	Davis	0.662	0.6620	0.7536	0.662	0.9848	0.996	0.1164
12	Winner21	0.662	0.6630	0.7529	0.742	0.9218	0.948	0.0741
13	Fool Me Once	0.661	0.6620	0.7489	0.662	0.9970	0.999	0.1191
14	Fortress4	0.552	0.5520	0.7467	0.707	1.0000	1.000	0.1676
15	Retaliate 3	0.662	0.6620	0.7448	0.662	0.9538	0.986	0.1032
16	EvolvedLookerUp1_1_1	0.662	0.6620	0.7422	0.662	0.9792	0.998	0.1062

**Table 9:** Top resistors for *N* = 7.

	Player	Min	5th %	Mean	Median	95th %	Max	Std
1	CS	0.858	0.9560	0.9765	0.981	1.000	1.000	0.0203
2	**TF1**	0.866	0.9521	0.9714	0.979	1.000	1.000	0.0207
3	**TF2**	0.840	0.9423	0.9677	0.976	0.998	1.000	0.0239
4	Predator	0.741	0.9478	0.9677	0.970	1.000	1.000	0.0367
5	Handshake	0.448	0.8261	0.9547	0.970	1.000	1.000	0.0848
6	Prober 4	0.837	0.8500	0.9540	0.955	1.000	1.000	0.0416
7	Winner21	0.858	0.8600	0.9392	0.956	0.996	0.999	0.0486
8	Hard Prober	0.856	0.8560	0.9331	0.953	1.000	1.000	0.0521
9	Fortress4	0.829	0.8290	0.9255	0.925	1.000	1.000	0.0653
10	Grudger	0.858	0.8580	0.9198	0.858	1.000	1.000	0.0642
11	**TF3**	0.858	0.8580	0.9189	0.858	1.000	1.000	0.0638
12	Davis	0.858	0.8580	0.9186	0.858	1.000	1.000	0.0633
13	Ripoff	0.856	0.8560	0.9183	0.922	0.986	0.988	0.0484
14	Tester	0.856	0.8560	0.9176	0.921	0.986	0.988	0.0486
15	MEM2	0.858	0.8580	0.9165	0.858	1.000	1.000	0.0636
16	Retaliate 3	0.858	0.8580	0.9161	0.858	0.999	1.000	0.0619

**Table 10:** Top resistors for *N* = 14.

	Player	Min	5th %	Mean	Median	95th %	Max	Std
1	CS	0.921	0.9970	0.9984	1.000	1.0	1.0	0.0062
2	**TF1**	0.938	0.9950	0.9973	0.999	1.0	1.0	0.0069
3	**TF2**	0.925	0.9820	0.9949	0.996	1.0	1.0	0.0104
4	Predator	0.836	0.9912	0.9941	0.999	1.0	1.0	0.0212
5	Prober 4	0.895	0.9110	0.9863	0.996	1.0	1.0	0.0250
6	Handshake	0.514	0.9131	0.9812	0.999	1.0	1.0	0.0743
7	Winner21	0.921	0.9210	0.9778	0.996	1.0	1.0	0.0310
8	Hard Prober	0.916	0.9160	0.9731	0.995	1.0	1.0	0.0327
9	Fortress4	0.929	0.9290	0.9726	0.981	1.0	1.0	0.0287
10	Ripoff	0.919	0.9190	0.9669	0.978	1.0	1.0	0.0318
11	Tester	0.919	0.9190	0.9662	0.977	1.0	1.0	0.0320
12	Grudger	0.921	0.9210	0.9592	0.921	1.0	1.0	0.0390
13	**TF3**	0.921	0.9210	0.9589	0.921	1.0	1.0	0.0388
14	Davis	0.921	0.9210	0.9588	0.921	1.0	1.0	0.0387
15	Retaliate 3	0.921	0.9210	0.9580	0.921	1.0	1.0	0.0383
16	Retaliate	0.921	0.9210	0.9576	0.921	1.0	1.0	0.0382

Interestingly none of these strategies are stochastic: this is explained by the value of not provoking typically cooperative opponent strategies with speculative defections. This includes opponents using the same strategy. Acting stochastically increases the chance of reducing the score of individuals of the same type in a Moran process. However it is possible to design a strategy with a stochastic or error-correcting handshake that is an excellent resistor even in noisy environments [16].

There are only two new strategies that appear in the top ranks for *x*_*N*−1_: TF1 and TF2. These two strategies are with CS the strongest resistors. They all have handshakes, and whilst the handshakes of CS and Handshake (which ranks highly for the smaller values of *N*) were programmed, the handshakes of TF1 and TF2 evolved without any priming.

As described previously the strategies trained with the payoff maximizing objective are among the best invaders in the library however they are not as resistant to invasion as the strategies trained using a Moran objective function. These strategies include trained finite state machine strategies, but they do not appear to have handshaking mechanisms. Therefore it is reasonable to conclude that the objective function is the cause of the emergence of handshaking mechanisms. More specifically, TF1 and TF2 evolved handshakes for high invasion resistance. TF3 is a better total payoff maximizer which makes it a better invader along with the strategies trained to maximize total payoff since successful fitness proportionate selection is necessary for invasion. Training with an objective with initial population mix other than (*N*/2, *N*/2) may favor invasion or resistance.

The payoff maximizing strategies typically will not defect before the opponent’s first defection, possibly because the training strategy collection contains some strategies such as Grudger and Fool Me Once that retaliate harshly by defecting for the remainder of the match if the opponent has more than a small number of cumulative defections. Paradoxically for handshaking strategies it is advantageous to defect (as a signal) in order to achieve mutual cooperation with opponents using the same strategy but not with other opponents. Nevertheless an evolutionary process is able to tunnel through the costs and risks associated with early defections to find more optimal solutions, so it is not surprising in hindsight that handshaking strategies emerge from the evolutionary training process.

A handshake requires at least one defection and there is selective pressure to defect as few times as possible to achieve the self-recognition mechanism. It is also unwise to defect on the first move as some strategies additionally retaliate in response to first round defections. So the handshakes used by TF1, TF2, and CS are in some sense optimal.

It is evident through the work presented that performance of strategies not only depends on the initial population distribution but also that there seems to be a difference depending on whether or not *N* > 2. This will be explored further in the next section, looking not only at *x*_1_ and *x*_*N*−1_ but also considering *x*_*N*/2_.

#### The effect of population size

**Fig 9:**
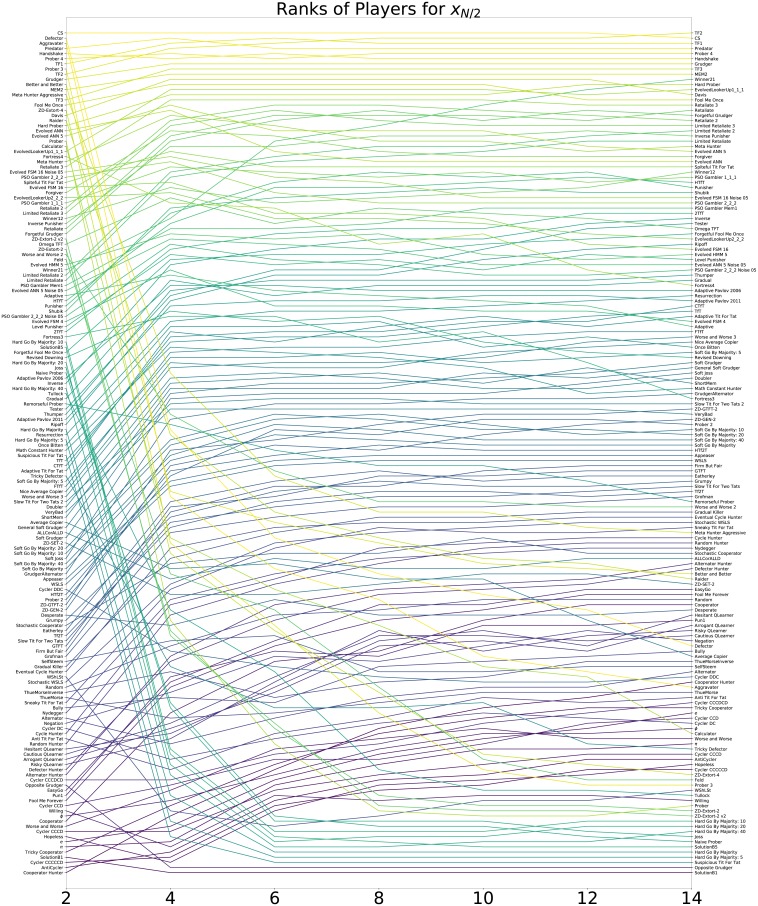
Fixation ranks of all strategies according to *x*_*N*/2_ for different population sizes.

Fig 9 complements Figs 7 and 8 showing the ranks of each strategy for all considered even values of *N* according to mean *x*_*N*/2_.

Tables 11, 12 and 13 show the ranks for a selection of strategies:

- The strategies that ranked highly for *N* = 2;
- The strategies that ranked highly for *N* = 14;
- The zero determinant strategies.

**Table 11:** Invasion: Fixation ranks of a few selected strategies according to *x*_1_ for different population sizes.

Size	2	3	4	5	6	7	8	9	10	11	12	13	14
CS	1	1	2	11	9	11	13	21	16	22	17	25	23
Defector	2	43	80	91	89	87	87	103	97	105	94	103	101
Aggravater	3	50	89	99	102	103	108	113	114	115	115	116	117
Predator	4	8	24	35	28	33	31	43	36	43	34	45	35
Handshake	5	17	40	46	43	46	46	49	48	49	47	50	49
Evolved FSM 16	31	11	6	2	1	1	1	1	1	1	1	1	1
PSO Gambler 2_2_2	29	14	10	6	4	2	2	2	2	2	2	2	2
EvolvedLookerUp2_2_2	33	18	11	9	10	6	6	5	3	5	3	3	3
Evolved ANN	20	10	8	7	8	5	3	3	4	3	4	4	4
Evolved ANN 5	21	9	7	8	7	4	5	4	5	4	5	5	5
**TF1**	7	13	33	38	30	39	42	46	42	46	41	46	46
**TF2**	9	19	29	33	19	28	29	38	27	34	26	32	30
**TF3**	14	4	5	5	6	9	11	11	12	14	13	13	16
ZD-Extort-4	16	81	107	120	135	136	142	140	142	142	144	144	145
ZD-Extort-2 v2	41	105	126	140	152	152	153	152	153	153	153	152	153
ZD-Extort-2	43	107	125	139	151	151	152	153	152	152	152	153	152
ZD-SET-2	100	111	117	117	122	127	131	128	131	131	130	132	131
ZD-GTFT-2	112	92	82	80	81	82	84	72	81	71	78	72	70
ZD-GEN-2	113	96	87	83	85	88	90	82	87	82	86	83	91

**Table 12:** Resistance: Fixation ranks of a few selected strategies according to *x*_*N*−1_ for different population sizes.

Size	2	3	4	5	6	7	8	9	10	11	12	13	14
CS	1	1	1	1	1	1	1	1	1	1	1	1	1
Defector	2	29	55	79	94	97	98	98	102	101	103	100	102
Aggravater	3	42	71	97	101	106	107	111	113	113	116	115	115
Predator	4	2	3	3	3	4	4	4	4	4	4	4	4
Handshake	5	4	5	5	5	5	5	6	6	6	6	6	6
**TF1**	7	3	2	2	2	2	2	2	2	2	2	2	2
**TF2**	10	5	4	4	4	3	3	3	3	3	3	3	3
Prober 4	6	6	6	6	6	6	6	5	5	5	5	5	5
**TF3**	13	9	10	11	11	11	13	14	13	13	13	13	13
ZD-Extort-4	19	68	98	106	108	114	115	115	118	118	117	118	117
ZD-Extort-2 v2	49	98	111	121	123	124	124	130	130	132	134	132	134
ZD-Extort-2	50	97	112	123	124	125	123	126	131	131	132	133	133
ZD-SET-2	108	105	104	104	103	103	100	100	101	99	98	98	98
ZD-GTFT-2	112	95	88	84	75	72	71	73	71	71	67	68	68
ZD-GEN-2	114	96	89	86	77	75	72	74	72	72	68	69	69

**Table 13:** Ranks of a few selected strategies according to *x*_*N*/2_ for different population sizes.

Size	2	4	6	8	10	12	14
CS	1	1	1	1	1	1	2
Defector	2	78	99	106	110	113	120
Aggravater	3	91	105	111	122	125	128
Predator	4	2	4	4	4	4	4
Handshake	5	6	5	6	6	6	6
**TF2**	9	4	3	2	2	2	1
**TF1**	7	3	2	3	3	3	3
Prober 4	6	5	6	5	5	5	5
**TF3**	14	8	8	8	8	8	8
ZD-Extort-4	16	102	117	129	141	143	145
ZD-Extort-2 v2	41	118	135	151	152	152	153
ZD-Extort-2	43	117	136	149	151	151	152
ZD-SET-2	100	110	110	108	106	106	108
ZD-GTFT-2	112	82	80	77	75	75	74
ZD-GEN-2	113	85	81	82	79	77	76

The results for *x*_*N*/2_ show similarities to the results for *x*_*N*−1_ and in particular TF1, TF2 and TF3 ranked first, third and eighth. This is to be expected since, as described previously these strategies were trained in an initial population of (*N*/2, *N*/2) individuals.

For all starting populations *i* ∈ {1, *N*/2, *N* − 1} the ranks of strategies are relatively stable across the different values of *N* > 2 however for *N* = 2 there is a distinct difference. This highlights that there is little that can be inferred about the evolutionary performance of a strategy in a large population from its performance in a small population. This is confirmed by the performance of the zero determinant strategies: while some do rank relatively highly for *N* = 2 (ZD-Extort-4 has rank 16) this rank does not translate to larger populations.

**Fig 10:**
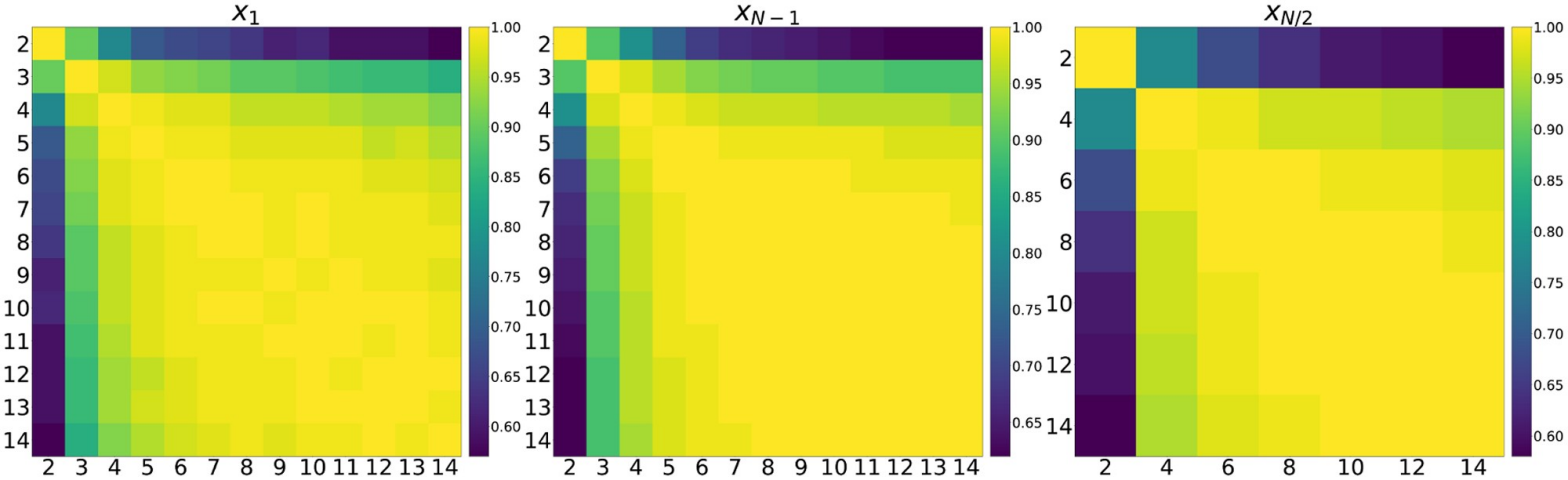
Heatmap of correlation coefficients of rankings by population size.

Fig 10 shows the correlation coefficients of the ranks of strategies in differing population size. How well a strategy performs in any Moran process for *N* > 2 has low correlation with the performance for *N* = 2. This illustrates why the strong performance of zero determinant strategies predicted in [4] does not extend to larger populations. This was discussed theoretically in [18] and observed empirically in these simulations.

## Discussion

Training strategies to excel at the Moran process leads to the evolution of cooperation, but only with like individuals in the case of TF1 and TF2. This may have significant implications for various biological and social phenomena such as human social interactions, particularly the evolution of ingroup/outgroup mechanisms and other sometimes costly rituals that reinforce group behavior.

While TF1 and TF2 are competent invaders, the best invaders in the study do not appear to employ strict handshakes, and are generally cooperative strategies. TF3, which does not use a handshake, is a better invader than TF1 and TF2 but not as good a resistor. Nevertheless it was the result of the same kind of training processes and is a better combined invader-resistor than the invaders that were trained previously to maximize payout. It is of interest to note that when trained in a payoff maximising criteria the finite state machines do not evolve to obtain a handshake, this highlights the importance of the evolutionary effect on this important mechanism.

The strategies trained to maximize payoff in head-to-head matches are generally cooperative and are effective invaders. Combined with the fact that handshaking strategies are stronger resistors, this suggests that while maximizing individual payoff can lead to the evolution of cooperation, these strategies are not the most evolutionarily stable in the long run. A strategy with a handshaking mechanism is still capable of invading and is more resistant to subsequent invasions. Moreover, the best resistor of the payoff maximally trained strategies (Evolved Looker Up 1_1_1), which always defects if the opponent defects in the first round, is effectively employing a one-shot handshake of C. Similarly, Grudger (also known as Grim), which emerged from training memory one strategies for the Moran process, also effectively employs a handshake of always cooperating, as it defects for the remainder of the match if the opponent ever defects.

The insights that payoff maximizers are better invaders and that handshakers are better resistors suggests that a strategy aware of the population distribution could choose to become a handshaker at a critical threshold and use a strategy better for invasion when in the minority. Information about the population distribution was not available to our strategies. Previous work has showed that strategies able to retain memory across matches can infer the population distribution and act in such a manner, resulting in a strategy effective at invasion and resistance [16].

We did not attempt other objective functions that may serve to select for both invasion and resistance better than training at a starting population of (*N*/2, *N*/2). Nevertheless our results suggest that there is not much room for improvement. Any handshake more sophisticated than always cooperate necessarily involves a defection. (A strategy with a handshake consisting of a long sequence of cooperations is effectively a grudger.) For TF3 or EvolvedLookerUp1_1_1 to become better resistors they need a longer or more strict handshake. But if this handshake involves a defection then likely the invasion ability is diminished for *N* > 2: the top invaders for larger *N* are nice strategies that do not defect before their opponents. This is because good invaders need to maximize match payoff to benefit from fitness proportionate selection, and so in the absence of a handshake mechanism, knowledge of the population distribution, or some identifying label on the opponent, a strategy must be generally cooperative. Aggressive strategies are only effective invaders for the smallest *N*, dropping dramatically in rank as the population size increases.

We did, however, attempt to evolve CS using finite state machines and lookup table based players, which resulted in some very similar strategies. In particular we evolved a lookup strategy that had a handshake of DC and played TFT with other players after a correct handshake while defecting otherwise, which is quite close in function to CS (full grudging is not possible with a lookup table of limited depth).

Finally we note that it may be possible to achieve similar results with smaller capacity finite state machine players.

## Conclusion

A detailed empirical analysis of 164 strategies of the IPD within a pairwise Moran process has been carried out. All (1642)=13,366 possible ordered pairs of strategies have been placed in a Moran process with different starting values allowing each strategy to attempt to invade the other. This is the largest such experiment carried out and has led to many insights.

When studying evolutionary processes it is vital to consider *N* > 2 since results for *N* = 2 cannot be used to extrapolate performance in larger populations. This was shown both observationally but also by considering the correlation of the ranks in different population sizes.

Memory one strategies do not perform as well as longer memory strategies in general in this study. Several longer memory strategies were high performers for invasion, particularly the strategies which have been trained using a number of reinforcement learning algorithms. Interestingly they have been trained to perform well in tournaments and not Moran processes specifically. In some cases these strategies utilize all the history of play (the neural network strategies and the lookup table strategies, the latter using the first round and some number of trailing rounds).

There are no memory one strategies in the top 5 performing strategies for *N* > 3. Training memory-one strategies specifically for the Moran process typically led to Grudger / Grim, a memory-one strategy with four-vector (1, 0, 0, 0). It appears to be the best resistor of the memory-one strategies. The highest performing memory-one strategy for invasion is PSO Gambler Mem 1, training to maximize total payout, which has four-vector (1, 0.52173487, 0, 0.12050939). For comparison, training for maximum score difference between the player and the opponent resulted in a strategy nearly the same as Grudger, with four-vector (0.9459, 0, 0, 0) (not included in the study).

**Fig 11:**
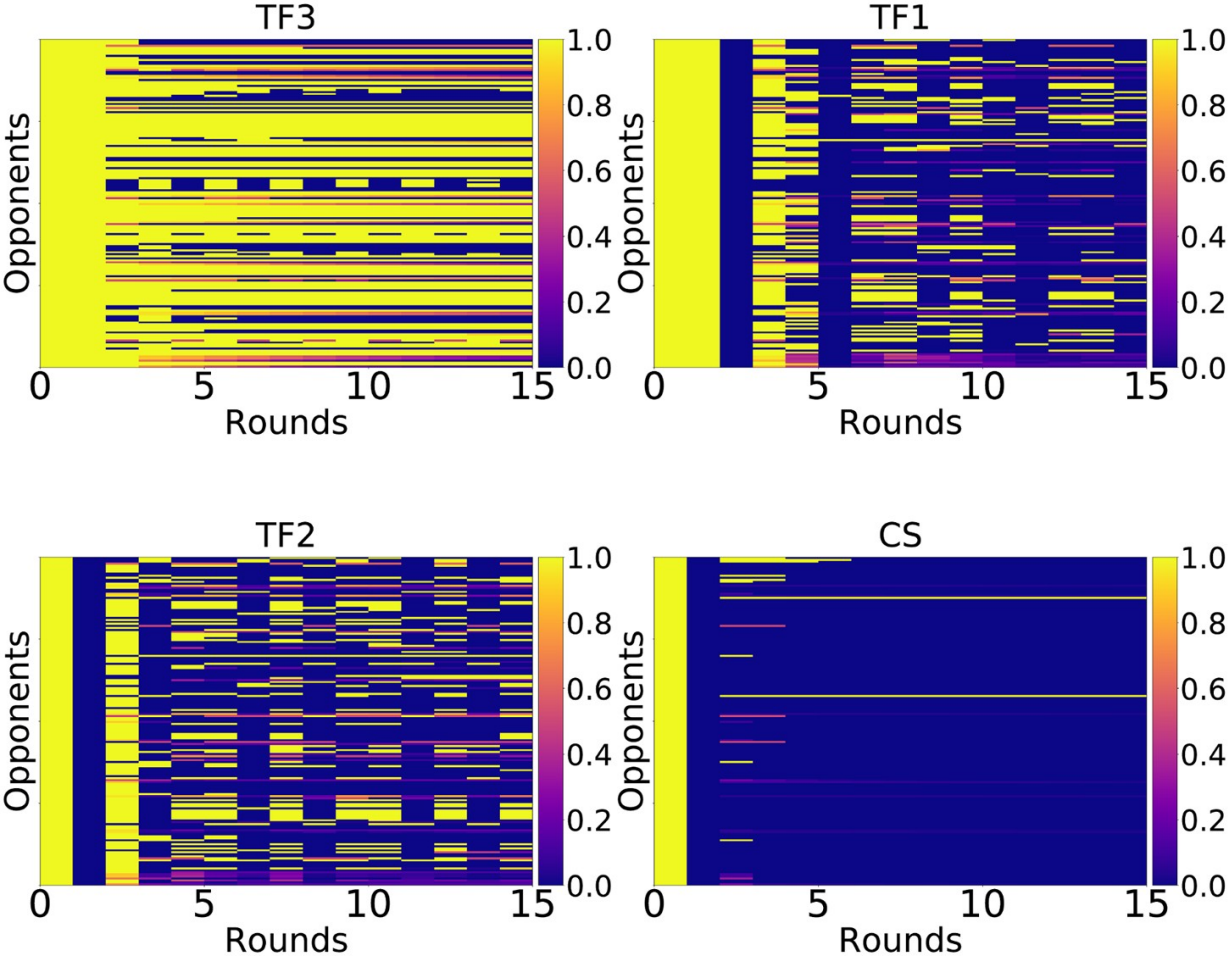
Cooperation rate per round (over 10000 repetitions). Rows correspond to all the strategies considered in this work (ordered alphabetically by name). Columns correspond to round of an IPD match.

One of the major findings discussed, is the ability of strategies with a handshake mechanism to resist invasion. This was not only revealed for CS (a human designed strategy) but also for two FSM strategies (TF1 and TF2) specifically trained through an evolutionary process. In these two cases, the handshake mechanism was a product of the evolutionary process. Fig 11 shows the cooperation rate of TF1, TF2, TF3 and CS for each round of a match against all the opponents in this study. This corresponds to the fraction of cooperation played by that strategy observed in a given round (out of the first 15) where each matchup is repeated 10000 times to obtain the mean.

While TF3 does not have a strict handshake mechanism it is clear that all these strategies start a match by cooperating. It is then evident that TF3 cooperates more than the other strategies thus explaining the difference in performance. It is also clear that CS only cooperates with itself and Handshake: it is a very aggressive strategy.

These findings are important for the ongoing understanding of population dynamics and offer evidence for some of the shortcomings of low memory which has started to be recognised by the community [17].

All source code for this work has been written in a sustainable manner: it is open source, under version control and tested which ensures that all results can be reproduced [30–32]. The raw data as well as the processed data has also been properly archived and can be found at [26].

There are many opportunities to build on this work. In particular, an analysis of the effect of noise should offer insights regarding the stability of the findings, particularly for the handshaking strategies. They may be less dominant for larger amounts of noise since the handshaking mechanisms may become brittle. There are many other variations to explore including populations with more than one type, spatial structure, and mutation.

One final point to recognise: the large set of strategies used here does not in itself constitute an authoritative set. Whilst it is not only large but also very diverse, the results (and rankings) presented might change given a different set of strategies. A further piece of work could look at subgroups of strategies and how they fair against other subgroups. Note that because of the open nature of the work here (not only is the source code archived but so is the data) this and any other further analysis is possible to carry out.

## Appendix: List of players

1. *ϕ*—*Deterministic*—*Memory depth*: ∞. [8]
2. *π*—*Deterministic*—*Memory depth*: ∞. [8]
3. *e*—*Deterministic*—*Memory depth*: ∞. [8]
4. ALLCorALLD—*Stochastic*—*Memory depth*: 1. [8]
5. Adaptive—*Deterministic*—*Memory depth*: ∞. [36]
6. Adaptive Pavlov 2006—*Deterministic*—*Memory depth*: ∞. [37]
7. Adaptive Pavlov 2011—*Deterministic*—*Memory depth*: ∞. [33]
8. Adaptive Tit For Tat: 0.5—*Deterministic*—*Memory depth*: ∞. [38]
9. Aggravater—*Deterministic*—*Memory depth*: ∞. [8]
10. Alternator—*Deterministic*—*Memory depth*: 1. [39, 40]
11. Alternator Hunter—*Deterministic*—*Memory depth*: ∞. [8]
12. Anti Tit For Tat—*Deterministic*—*Memory depth*: 1. [41]
13. AntiCycler—*Deterministic*—*Memory depth*: ∞. [8]
14. Appeaser—*Deterministic*—*Memory depth*: ∞. [8]
15. Arrogant QLearner—*Stochastic*—*Memory depth*: ∞. [8]
16. Average Copier—*Stochastic*—*Memory depth*: ∞. [8]
17. Better and Better—*Stochastic*—*Memory depth*: ∞. [27]
18. Bully—*Deterministic*—*Memory depth*: 1. [42]
19. Calculator—*Stochastic*—*Memory depth*: ∞. [27]
20. Cautious QLearner—*Stochastic*—*Memory depth*: ∞. [8]
21. CollectiveStrategy(**CS**)—*Deterministic*—*Memory depth*: ∞. [28]
22. Contrite Tit For Tat(**CTfT**)—*Deterministic*—*Memory depth*: 3. [43]
23. Cooperator—*Deterministic*—*Memory depth*: 0. [4, 39, 40]
24. Cooperator Hunter—*Deterministic*—*Memory depth*: ∞. [8]
25. Cycle Hunter—*Deterministic*—*Memory depth*: ∞. [8]
26. Cycler CCCCCD—*Deterministic*—*Memory depth*: 5. [8]
27. Cycler CCCD—*Deterministic*—*Memory depth*: 3. [8]
28. Cycler CCCDCD—*Deterministic*—*Memory depth*: 5. [8]
29. Cycler CCD—*Deterministic*—*Memory depth*: 2. [40]
30. Cycler DC—*Deterministic*—*Memory depth*: 1. [8]
31. Cycler DDC—*Deterministic*—*Memory depth*: 2. [40]
32. Davis: 10—*Deterministic*—*Memory depth*: ∞. [2]
33. Defector—*Deterministic*—*Memory depth*: 0. [4, 39, 40]
34. Defector Hunter—*Deterministic*—*Memory depth*: ∞. [8]
35. Desperate—*Stochastic*—*Memory depth*: 1. [44]
36. Doubler—*Deterministic*—*Memory depth*: ∞. [27]
37. EasyGo—*Deterministic*—*Memory depth*: ∞. [27, 36]
38. Eatherley—*Stochastic*—*Memory depth*: ∞. [45]
39. Eventual Cycle Hunter—*Deterministic*—*Memory depth*: ∞. [8]
40. Evolved ANN—*Deterministic*—*Memory depth*: ∞. [8]
41. Evolved ANN 5—*Deterministic*—*Memory depth*: ∞. [8]
42. Evolved ANN 5 Noise 05—*Deterministic*—*Memory depth*: ∞. [8]
43. Evolved FSM 16—*Deterministic*—*Memory depth*: 16—*Number of states*: 14. [8]
44. Evolved FSM 16 Noise 05—*Deterministic*—*Memory depth*: 16—*Number of states*: 14. [8]
45. Evolved FSM 4—*Deterministic*—*Memory depth*: 4—*Number of states*: 4. [8]
46. Evolved HMM 5—*Stochastic*—*Memory depth*: 5. [8]
47. EvolvedLookerUp1_1_1—*Deterministic*—*Memory depth*: ∞. [8]
48. EvolvedLookerUp2_2_2—*Deterministic*—*Memory depth*: ∞. [8]
49. FSM Player: [(0, ‘C’, 0, ‘C’), (0, ‘D’, 3, ‘C’), (1, ‘C’, 5, ‘D’), (1, ‘D’, 0, ‘C’), (2, ‘C’, 3, ‘C’), (2, ‘D’, 2, ‘D’), (3, ‘C’, 4, ‘D’), (3, ‘D’, 6, ‘D’), (4, ‘C’, 3, ‘C’), (4, ‘D’, 1, ‘D’), (5, ‘C’, 6, ‘C’), (5, ‘D’, 3, ‘D’), (6, ‘C’, 6, ‘D’), (6, ‘D’, 6, ‘D’), (7, ‘C’, 7, ‘D’), (7, ‘D’, 5, ‘C’)], 0, C(**TF3**)—*Deterministic*—*Memory depth*: ∞—*Number of states*: 8.
50. FSM Player: [(0, ‘C’, 13, ‘D’), (0, ‘D’, 12, ‘D’), (1, ‘C’, 3, ‘D’), (1, ‘D’, 4, ‘D’), (2, ‘C’, 14, ‘D’), (2, ‘D’, 9, ‘D’), (3, ‘C’, 0, ‘C’), (3, ‘D’, 1, ‘D’), (4, ‘C’, 1, ‘D’), (4, ‘D’, 2, ‘D’), (5, ‘C’, 12, ‘C’), (5, ‘D’, 6, ‘C’), (6, ‘C’, 1, ‘C’), (6, ‘D’, 14, ‘D’), (7, ‘C’, 12, ‘D’), (7, ‘D’, 2, ‘D’), (8, ‘C’, 7, ‘D’), (8, ‘D’, 9, ‘D’), (9, ‘C’, 8, ‘D’), (9, ‘D’, 0, ‘D’), (10, ‘C’, 2, ‘C’), (10, ‘D’, 15, ‘C’), (11, ‘C’, 7, ‘D’), (11, ‘D’, 13, ‘D’), (12, ‘C’, 3, ‘C’), (12, ‘D’, 8, ‘D’), (13, ‘C’, 7, ‘C’), (13, ‘D’, 10, ‘D’), (14, ‘C’, 10, ‘D’), (14, ‘D’, 7, ‘D’), (15, ‘C’, 15, ‘C’), (15, ‘D’, 11, ‘D’)], 0, C(**TF2**)—*Deterministic*—*Memory depth*: ∞—*Number of states*: 16.
51. FSM Player: [(0, ‘C’, 7, ‘C’), (0, ‘D’, 1, ‘C’), (1, ‘C’, 11, ‘D’), (1, ‘D’, 11, ‘D’), (2, ‘C’, 8, ‘D’), (2, ‘D’, 8, ‘C’), (3, ‘C’, 3, ‘C’), (3, ‘D’, 12, ‘D’), (4, ‘C’, 6, ‘C’), (4, ‘D’, 3, ‘C’), (5, ‘C’, 11, ‘C’), (5, ‘D’, 8, ‘D’), (6, ‘C’, 13, ‘D’), (6, ‘D’, 14, ‘C’), (7, ‘C’, 4, ‘D’), (7, ‘D’, 2, ‘D’), (8, ‘C’, 14, ‘D’), (8, ‘D’, 8, ‘D’), (9, ‘C’, 0, ‘C’), (9, ‘D’, 10, ‘D’), (10, ‘C’, 8, ‘C’), (10, ‘D’, 15, ‘C’), (11, ‘C’, 6, ‘D’), (11, ‘D’, 5, ‘D’), (12, ‘C’, 6, ‘D’), (12, ‘D’, 9, ‘D’), (13, ‘C’, 9, ‘D’), (13, ‘D’, 8, ‘D’), (14, ‘C’, 8, ‘D’), (14, ‘D’, 13, ‘D’), (15, ‘C’, 4, ‘C’), (15, ‘D’, 5, ‘C’)], 0, C(**TF1**)—*Deterministic*—*Memory depth*: ∞—*Number of states*: 16.
52. Feld: 1.0, 0.5, 200—*Stochastic*—*Memory depth*: 200. [2]
53. Firm But Fair—*Stochastic*—*Memory depth*: 1. [46]
54. Fool Me Forever—*Deterministic*—*Memory depth*: ∞. [8]
55. Fool Me Once—*Deterministic*—*Memory depth*: ∞. [8]
56. Forgetful Fool Me Once: 0.05—*Stochastic*—*Memory depth*: ∞. [8]
57. Forgetful Grudger—*Deterministic*—*Memory depth*: 10. [8]
58. Forgiver—*Deterministic*—*Memory depth*: ∞. [8]
59. Forgiving Tit For Tat(**FTfT**)—*Deterministic*—*Memory depth*: ∞. [8]
60. Fortress3—*Deterministic*—*Memory depth*: 3—*Number of states*: 3. [21]
61. Fortress4—*Deterministic*—*Memory depth*: 4—*Number of states*: 4. [21]
62. GTFT: 0.33—*Stochastic*—*Memory depth*: 1. [22, 47]
63. General Soft Grudger: n = 1,d = 4,c = 2—*Deterministic*—*Memory depth*: ∞. [8]
64. Gradual—*Deterministic*—*Memory depth*: ∞. [48]
65. Gradual Killer: (‘D’, ‘D’, ‘D’, ‘D’, ‘D’, ‘C’, ‘C’)—*Deterministic*—*Memory depth*: ∞. [27]
66. Grofman—*Stochastic*—*Memory depth*: ∞. [2]
67. Grudger—*Deterministic*—*Memory depth*: 1. [2, 36, 44, 48, 49]
68. GrudgerAlternator—*Deterministic*—*Memory depth*: ∞. [27]
69. Grumpy: Nice, 10, -10—*Deterministic*—*Memory depth*: ∞. [8]
70. Handshake—*Deterministic*—*Memory depth*: ∞. [10]
71. Hard Go By Majority—*Deterministic*—*Memory depth*: ∞. [40]
72. Hard Go By Majority: 10—*Deterministic*—*Memory depth*: 10. [8]
73. Hard Go By Majority: 20—*Deterministic*—*Memory depth*: 20. [8]
74. Hard Go By Majority: 40—*Deterministic*—*Memory depth*: 40. [8]
75. Hard Go By Majority: 5—*Deterministic*—*Memory depth*: 5. [8]
76. Hard Prober—*Deterministic*—*Memory depth*: ∞. [27]
77. Hard Tit For 2 Tats(**HTf2T**)—*Deterministic*—*Memory depth*: 3. [50]
78. Hard Tit For Tat(**HTfT**)—*Deterministic*—*Memory depth*: 3. [51]
79. Hesitant QLearner—*Stochastic*—*Memory depth*: ∞. [8]
80. Hopeless—*Stochastic*—*Memory depth*: 1. [44]
81. Inverse—*Stochastic*—*Memory depth*: ∞. [8]
82. Inverse Punisher—*Deterministic*—*Memory depth*: ∞. [8]
83. Joss: 0.9—*Stochastic*—*Memory depth*: 1. [2, 50]
84. Level Punisher—*Deterministic*—*Memory depth*: ∞. [52]
85. Limited Retaliate 2: 0.08, 15—*Deterministic*—*Memory depth*: ∞. [8]
86. Limited Retaliate 3: 0.05, 20—*Deterministic*—*Memory depth*: ∞. [8]
87. Limited Retaliate: 0.1, 20—*Deterministic*—*Memory depth*: ∞. [8]
88. MEM2—*Deterministic*—*Memory depth*: ∞. [14]
89. Math Constant Hunter—*Deterministic*—*Memory depth*: ∞. [8]
90. Meta Hunter Aggressive: 7 players—*Deterministic*—*Memory depth*: ∞. [8]
91. Meta Hunter: 6 players—*Deterministic*—*Memory depth*: ∞. [8]
92. Naive Prober: 0.1—*Stochastic*—*Memory depth*: 1. [36]
93. Negation—*Stochastic*—*Memory depth*: 1. [51]
94. Nice Average Copier—*Stochastic*—*Memory depth*: ∞. [8]
95. Nydegger—*Deterministic*—*Memory depth*: 3. [2]
96. Omega TFT: 3, 8—*Deterministic*—*Memory depth*: ∞. [37]
97. Once Bitten—*Deterministic*—*Memory depth*: 12. [8]
98. Opposite Grudger—*Deterministic*—*Memory depth*: ∞. [8]
99. PSO Gambler 1_1_1—*Stochastic*—*Memory depth*: ∞. [8]
100. PSO Gambler 2_2_2—*Stochastic*—*Memory depth*: ∞. [8]
101. PSO Gambler 2_2_2 Noise 05—*Stochastic*—*Memory depth*: ∞. [8]
102. PSO Gambler Mem1—*Stochastic*—*Memory depth*: 1. [8]
103. Predator—*Deterministic*—*Memory depth*: 9—*Number of states*: 9. [21]
104. Prober—*Deterministic*—*Memory depth*: ∞. [36]
105. Prober 2—*Deterministic*—*Memory depth*: ∞. [27]
106. Prober 3—*Deterministic*—*Memory depth*: ∞. [27]
107. Prober 4—*Deterministic*—*Memory depth*: ∞. [27]
108. Pun1—*Deterministic*—*Memory depth*: 2—*Number of states*: 2. [21]
109. Punisher—*Deterministic*—*Memory depth*: ∞. [8]
110. Raider—*Deterministic*—*Memory depth*: 3—*Number of states*: 4. [53]
111. Random Hunter—*Deterministic*—*Memory depth*: ∞. [8]
112. Random: 0.5—*Stochastic*—*Memory depth*: 0. [2, 38]
113. Remorseful Prober: 0.1—*Stochastic*—*Memory depth*: 2. [36]
114. Resurrection—*Deterministic*—*Memory depth*: 1. [52]
115. Retaliate 2: 0.08—*Deterministic*—*Memory depth*: ∞. [8]
116. Retaliate 3: 0.05—*Deterministic*—*Memory depth*: ∞. [8]
117. Retaliate: 0.1—*Deterministic*—*Memory depth*: ∞. [8]
118. Revised Downing: True—*Deterministic*—*Memory depth*: ∞. [2]
119. Ripoff—*Deterministic*—*Memory depth*: 2—*Number of states*: 3. [54]
120. Risky QLearner—*Stochastic*—*Memory depth*: ∞. [8]
121. SelfSteem—*Stochastic*—*Memory depth*: ∞. [55]
122. ShortMem—*Deterministic*—*Memory depth*: 10. [55]
123. Shubik—*Deterministic*—*Memory depth*: ∞. [2]
124. Slow Tit For Two Tats—*Deterministic*—*Memory depth*: 2. [8]
125. Slow Tit For Two Tats 2—*Deterministic*—*Memory depth*: 2. [27]
126. Sneaky Tit For Tat—*Deterministic*—*Memory depth*: ∞. [8]
127. Soft Go By Majority—*Deterministic*—*Memory depth*: ∞. [39, 40]
128. Soft Go By Majority: 10—*Deterministic*—*Memory depth*: 10. [8]
129. Soft Go By Majority: 20—*Deterministic*—*Memory depth*: 20. [8]
130. Soft Go By Majority: 40—*Deterministic*—*Memory depth*: 40. [8]
131. Soft Go By Majority: 5—*Deterministic*—*Memory depth*: 5. [8]
132. Soft Grudger—*Deterministic*—*Memory depth*: 6. [36]
133. Soft Joss: 0.9—*Stochastic*—*Memory depth*: 1. [27]
134. SolutionB1—*Deterministic*—*Memory depth*: 3—*Number of states*: 3. [56]
135. SolutionB5—*Deterministic*—*Memory depth*: 5—*Number of states*: 6. [56]
136. Spiteful Tit For Tat—*Deterministic*—*Memory depth*: ∞. [27]
137. Stochastic Cooperator—*Stochastic*—*Memory depth*: 1. [18]
138. Stochastic WSLS: 0.05—*Stochastic*—*Memory depth*: 1. [8]
139. Suspicious Tit For Tat—*Deterministic*—*Memory depth*: 1. [41, 48]
140. Tester—*Deterministic*—*Memory depth*: ∞. [45]
141. ThueMorse—*Deterministic*—*Memory depth*: ∞. [8]
142. ThueMorseInverse—*Deterministic*—*Memory depth*: ∞. [8]
143. Thumper—*Deterministic*—*Memory depth*: 2—*Number of states*: 2. [54]
144. Tit For 2 Tats(**Tf2T**)—*Deterministic*—*Memory depth*: 2. [39]
145. Tit For Tat(**TfT**)—*Deterministic*—*Memory depth*: 1. [2]
146. Tricky Cooperator—*Deterministic*—*Memory depth*: 10. [8]
147. Tricky Defector—*Deterministic*—*Memory depth*: ∞. [8]
148. Tullock: 11—*Stochastic*—*Memory depth*: 11. [2]
149. Two Tits For Tat(**2TfT**)—*Deterministic*—*Memory depth*: 2. [39]
150. VeryBad—*Deterministic*—*Memory depth*: ∞. [55]
151. Willing—*Stochastic*—*Memory depth*: 1. [44]
152. Win-Shift Lose-Stay: D(**WShLSt**)—*Deterministic*—*Memory depth*: 1. [36]
153. Win-Stay Lose-Shift: C(**WSLS**)—*Deterministic*—*Memory depth*: 1. [47, 50, 57]
154. Winner12—*Deterministic*—*Memory depth*: 2. [58]
155. Winner21—*Deterministic*—*Memory depth*: 2. [58]
156. Worse and Worse—*Stochastic*—*Memory depth*: ∞. [27]
157. Worse and Worse 2—*Stochastic*—*Memory depth*: ∞. [27]
158. Worse and Worse 3—*Stochastic*—*Memory depth*: ∞. [27]
159. ZD-Extort-2 v2: 0.125, 0.5, 1—*Stochastic*—*Memory depth*: 1. [59]
160. ZD-Extort-2: 0.1111111111111111, 0.5—*Stochastic*—*Memory depth*: 1. [50]
161. ZD-Extort-4: 0.23529411764705882, 0.25, 1—*Stochastic*—*Memory depth*: 1. [8]
162. ZD-GEN-2: 0.125, 0.5, 3—*Stochastic*—*Memory depth*: 1. [59]
163. ZD-GTFT-2: 0.25, 0.5—*Stochastic*—*Memory depth*: 1. [50]
164. ZD-SET-2: 0.25, 0.0, 2—*Stochastic*—*Memory depth*: 1. [59]

## Supporting information

S1 Fig The fixation probabilities *x*_1_ for *N* = 3. (EPS)

S2 Fig The fixation probabilities *x*_1_ for *N* = 4. (EPS)

S3 Fig The fixation probabilities *x*_1_ for *N* = 5. (EPS)

S4 Fig The fixation probabilities *x*_1_ for *N* = 6. (EPS)

S5 Fig The fixation probabilities *x*_1_ for *N* = 7. (EPS)

S6 Fig The fixation probabilities *x*_1_ for *N* = 8. (EPS)

S7 Fig The fixation probabilities *x*_1_ for *N* = 9. (EPS)

S8 Fig The fixation probabilities *x*_1_ for *N* = 10. (EPS)

S9 Fig The fixation probabilities *x*_1_ for *N* = 11. (EPS)

S10 Fig The fixation probabilities *x*_1_ for *N* = 12. (EPS)

S11 Fig The fixation probabilities *x*_1_ for *N* = 13. (EPS)

S12 Fig The fixation probabilities *x*_1_ for *N* = 14. (EPS)

S13 Fig The fixation probabilities *x*_*N*−1_ for *N* = 3. (EPS)

S14 Fig The fixation probabilities *x*_*N*−1_ for *N* = 4. (EPS)

S15 Fig The fixation probabilities *x*_*N*−1_ for *N* = 5. (EPS)

S16 Fig The fixation probabilities *x*_*N*−1_ for *N* = 6. (EPS)

S17 Fig The fixation probabilities *x*_*N*−1_ for *N* = 7. (EPS)

S18 Fig The fixation probabilities *x*_*N*−1_ for *N* = 8. (EPS)

S19 Fig The fixation probabilities *x*_*N*−1_ for *N* = 9. (EPS)

S20 Fig The fixation probabilities *x*_*N*−1_ for *N* = 10. (EPS)

S21 Fig The fixation probabilities *x*_*N*−1_ for *N* = 11. (EPS)

S22 Fig The fixation probabilities *x*_*N*−1_ for *N* = 12. (EPS)

S23 Fig The fixation probabilities *x*_*N*−1_ for *N* = 13. (EPS)

S24 Fig The fixation probabilities *x*_*N*−1_ for *N* = 14. (EPS)
